# Burnout Among Physicians With Disabilities

**DOI:** 10.1001/jamanetworkopen.2024.10701

**Published:** 2024-05-09

**Authors:** Lisa M. Meeks, Sarah S. Conrad, Zakia Nouri, Christopher J. Moreland, Zoie C. Sheets, Xiaochu Hu, Michael J. Dill

**Affiliations:** 1 Learning Health Sciences, University of Michigan Medical School, Ann Arbor; 2 Family Medicine, University of Michigan Medical School, Ann Arbor; 3 Workforce Studies, Association of American Medical Colleges, Washington, DC; 4 Dell Medical School, University of Texas at Austin, Austin; 5 Department of Medicine, University of Chicago, Chicago, Illinois; 6 Department of Pediatrics, University of Chicago, Chicago, Illinois

## Abstract

This survey study examines reported experiences of burnout, including emotional exhaustion and depersonalization, among physicians with disability.

## Introduction

Burnout among physicians and health care practitioners is a national crisis. It affects the health and well-being of physicians, health care costs, health care quality, and physician attrition.^1,2,3^ Mistreatment is a known correlate of burnout,^4^ and physicians with disabilities (PWDs), an integral part of the physician workforce, are at an increased risk for mistreatment, placing them at higher risk for burnout.^5^ Despite known stressors for this population, burnout in PWD has not been studied. To address this gap, we investigated the burnout experiences among PWDs in the US.

## Methods

We analyzed the Association of American Medical College’s 2022 National Sample Survey of Physicians (NSSP) data (eMethods in Supplement 1). This dataset contains information on nationally representative physicians (eMethods in Supplement 1) who completed the NSSP between May and November 2022 and consented for their data to be included in research. Physicians were asked whether they had a disability and, if so, to indicate their disability (ie, attention-deficit/hyperactivity disorder, chronic health issue, hearing impairment, learning disability, mobility disability, psychological disability, vision impairment, or other). The American Institutes for Research Institutional Review Board deemed this survey study exempt from review. We followed the AAPOR reporting guideline.

Burnout was measured using 2 dimensions from the Maslach Burnout Inventory: depersonalization and emotional exhaustion. Physicians used a 7-point scale (never, a few times a year or less, once a month or less, a few times a month, once a week, a few times a week, and every day) to report the frequency with which *I have become more callous toward people since I took this job* and *I feel burned out from my work*.

We ran 2 multivariate ordered logistic regressions to assess the likelihood of PWDs to experience emotional exhaustion and depersonalization, controlling for demographic variables (age group, gender identity, sexual orientation, marital and parental status, and race and ethnicity), workplace characteristics (work settings, weekly work hours, specialty group, academic affiliation, and time use), and international medical graduate status. We set significance levels at *P* < .05. Data analysis was performed with Stata SE/17 (StataCorp LLC).

## Results

A total of 5917 physicians (3722 males [62.9%], 2195 females [37.1%]; mean [SD] age, 51.98 [11.78] years) completed the NSSP. Of these physicians, 185 (3.1%) reported having a disability. The most frequently selected disabilities were chronic health (60 [32.4%]) and mobility (46 [24.9%]). For PWDs, the odds of reporting daily depersonalization were higher (adjusted odds ratio, 1.45; 95% CI, 1.11-1.91; *P* = .007) than for their peers (Table 1). Additionally, PWDs reported emotional exhaustion more frequently than physicians without disability, but the difference was not statistically significant (Table 2).

**Table 1.** Adjusted Odds Ratios (AORs) for Physicians Feeling Depersonalized^a^

Characteristics	AOR (95% CI)	*P* value
PWDs reporting daily depersonalization	1.45 (1.11-1.91)	.007
Gender identity		
Woman and transgender woman	1 [Reference]	
Man and transgender man	0.83 (0.75-0.93)	.001
Genderqueer or other^b^	0.91 (0.47-1.75)	.77
Race and ethnicity^c^		
Hispanic, Latino, or of Spanish origin	1 [Reference]	
American Indian or Alaskan Native	1.10 (0.30-4.12)	.88
Asian	0.99 (0.74-1.32)	.95
Black or African American	0.54 (0.36-0.82)	.003
Native Hawaiian or other Pacific Islander	1.59 (0.53-4.75)	.40
White	1.24 (0.93-1.64)	.14
Multiracial and other^d^	0.91 (0.63-1.30)	.60
Sexual orientation		
Heterosexual	0.86 (0.69-1.06)	.16
Age group, y		
≤35	1 [Reference]	
36-55	0.92 (0.77-1.11)	.40
56-75	0.41 (0.33-0.50)	<.001
≥76	0.17 (0.11-0.27)	<.001
Married	0.98 (0.86-1.12)	.80
Presence of children aged ≤5 y	1.11 (0.96-1.28)	.15
Working in hospitals^e^	0.98 (0.86-1.11)	.77
Specialty group		
Medical specialties	1 [Reference]	
Other^f^	1.22 (1.07-1.41)	.004
Primary care	1.08 (0.94-1.24)	.29
Surgery	1.00 (0.86-1.16)	>.99
Usual hours of work in a week	1.01 (1.00-1.01)	<.001
Academically affiliated	0.95 (0.85-1.06)	.36
Working in private setting^e^	0.81 (0.72-0.90)	<.001
Time use: mean % of total weekly work hours spent in the following activity:^g^		
Patient care	1.00 (1.00-1.00)	.14
Clinical documentation and communicating with insurance companies or other payers (via telephone, email, etc.)	1.01 (1.01-1.02)	<.001
Research	1.00 (0.99-1.00)	.25
Teaching	1.00 (0.99-1.01)	.62
Administration	1.00 (1.00-1.01)	.53
International medical graduate	0.62 (0.54-0.72)	<.001

Abbreviation: PWDs, physicians with disabilities.

^a^ Analyses of the 2022 Association of American Medical College (AAMC) National Sample Survey of Physicians data (N = 5536).

^b^ Other gender identity included agender and genderqueer/gender nonconforming, among others.

^c^ Race and ethnicity were secondary reported and merged from multiple AAMC surveys in which respondents self-reported race and ethnicity.

^d^ No further information was available.

^e^ Respondents described their current primary employment arrangement from a list and were allowed to choose more than 1.

^f^ Other specialty group included anesthesiology and psychiatry, among others.

^g^ Respondents were asked to give an estimate of the percentage of total weekly work hours they spent in each of the given activities; the total added to 100%.

**Table 2.** Adjusted Odds Ratios (AORs) for Physicians Feeling Emotional Exhaustion^a^

Characteristics	AOR (95% CI)	*P* value
PWDs reporting daily emotional exhaustion	1.19 (0.91-1.57)	.21
Gender identity		
Woman and transgender woman	1 [Reference]	
Man and transgender man	0.70 (0.63-0.78)	<.001
Genderqueer or other^b^	0.47 (0.23-0.95)	.04
Race and ethnicity^c^		
Hispanic, Latino, or of Spanish origin	1 [Reference]	
American Indian or Alaskan Native	3.15 (0.95-10.39)	.06
Asian	0.84 (0.63-1.11)	.22
Black or African American	0.70 (0.47-1.05)	.08
Native Hawaiian or other Pacific Islander	1.58 (0.52-4.78)	.42
White	1.07 (0.81-1.40)	.64
Multiracial and other^d^	1.00 (0.71-1.42)	>.99
Sexual orientation		
Heterosexual	0.82 (0.66-1.01)	.07
Age group, y		
≤35	1 [Reference]	
36-55	1.26 (1.05-1.51)	.01
56-75	0.76 (0.62-0.92)	.01
≥76	0.20 (0.13-0.31)	<.001
Married	0.97 (0.85-1.10)	.64
Presence of children aged ≤5 y	0.99 (0.86-1.13)	.85
Working in hospitals^e^	0.97 (0.85-1.09)	.58
Specialty group		
Medical specialties	1 [Reference]	
Other^f^	1.12 (0.98-1.28)	.11
Primary care	1.26 (1.10-1.44)	.001
Surgery	0.91 (0.78-1.06)	.21
Usual hours of work in a week	1.01 (1.01-1.02)	<.001
Academically affiliated	1.04 (0.93-1.16)	.53
Working in private setting^e^	0.79 (0.71-0.88)	<.001
Time use: mean % of total weekly work hours spent in the following activity:^g^		
Patient care	1.00 (1.00-1.00)	.12
Clinical documentation and communicating with insurance companies or other payers (via telephone or email)	1.01 (1.01-1.02)	<.001
Research	0.99 (0.99-1.00)	.06
Teaching	0.99 (0.98-1.00)	.01
Administration	1.00 (1.00-1.01)	.28
International medical graduate	0.72 (0.63-0.83)	<.001

Abbreviation: PWDs, physicians with disabilities.

^a^ Analyses of the 2022 Association of American Medical College (AAMC) National Sample Survey of Physicians data (N = 5546).

^b^ Other gender identity included agender and genderqueer/gender nonconforming, among others.

^c^ Race and ethnicity were secondary reported and merged from multiple AAMC surveys in which respondents self-reported race and ethnicity.

^d^ No further information was available.

^e^ Respondents described their current primary employment arrangement from a list and were allowed to choose more than 1.

^f^ Other specialty group included anesthesiology and psychiatry, among others.

^g^ Respondents were asked to give an estimate of the percentage of total weekly work hours they spent in each of the given activities; the total added to 100%.

## Discussion

Compared with peers, PWDs were significantly more likely to experience depersonalization at least once during the previous year, but not emotional exhaustion. This finding suggests PWDs have some protective qualities against exhaustion. However, it simultaneously amplifies growing concerns about the structural environments in which PWDs work, including lack of protections against mistreatment, harassment, and pay inequity.^5,6^

The sustainability of entering and remaining in the health care workforce without structural-level intervention is tenuous for PWDs. Health care systems must develop a multifaceted approach to decreasing mistreatment, increasing a sense of belonging, promoting pay equity, and ensuring psychological and physical safety for PWDs. Strategies may include antiableist training, sharing successes, pay equity evaluations, and robust reporting options for disability-related mistreatment.

Study limitations include the self-reported nature of survey data and potential underreporting of disability due to fear of stigma. Additionally, physicians with high levels of burnout may opt out of surveys.

Future research may include qualitative studies to identify factors in burnout among PWDs. Additionally, studies that investigate the effectiveness of mechanisms focused on mistreatment reduction, pay equity, increased access (including accommodations), and intent to leave the workforce would provide valuable information to initiatives for retaining this physician population.

## Notes

Supplement 1. **eMethods.** Notes on Sampling Methods

Supplement 2.
Data Sharing Statement
