# Supplementary material for: Burnout Among Physicians With Disabilities
Source: JAMA Netw Open. 2024 May 9;7(5):e2410701. doi: 10.1001/jamanetworkopen.2024.10701 (PMC11082676; doi:10.1001/jamanetworkopen.2024.10701)
Supplement: Supplement 1. — eMethods. Notes on Sampling Methods [file jamanetwopen-e2410701-s001.pdf]

Supplemental Online Content

Meeks LM, Conrad SS, Nouri Z, et al. Burnout among physicians with disabilities. *JAMA Netw Open*. 2024;7(5):e2410701. doi:10.1001/jamanetworkopen.2024.10701

eMethods. Notes on Sampling Methods

This supplemental material has been provided by the authors to give readers additional information about their work.

© 2024 Meeks LM et al. *JAMA Network Open*

eMethods. Notes on Sampling Methods

Overview

The 2022 National Sample Survey of Physicians (NSSP 2022) was conducted between May 10, 2022, and Nov 9, 2022, and the dataset consists of 5,917 active physicians in the U.S. The NSSP project was funded by The Association of American Medical Colleges (AAMC) and the survey content was designed by the Workforce Studies team at AAMC. The survey was conducted through an online survey fielded and managed by Toluna, following a sampling methodology provided by The University of Michigan Population Dynamics and Health Program. The NSSP 2022 project was reviewed and approved by the American Institutes for Research in the Behavioral Sciences Institutional Review Board (IRB00000436).

The NSSP 2022 is the second wave of the AAMC NSSP series. The first wave was conducted in 2019. (We refer to NSSP 2019 as Wave 1 and NSSP 2022 as Wave 2 throughout this document.) NSSP 2022 includes two groups of respondents: the repeating respondents from Wave 1 (N=2,429), and new invitees (N=3,488).

Sample Design and Probabilities of Selection

Overview

NSSP 2022 sampling followed a strategy developed by The University of Michigan Population Dynamics and Health Program team (PDHP). The data were collected in two phases: 1) all eligible respondents from the Wave 1 survey (N=4,418 of the 6,000 Wave 1 respondents, as 1,582 of Wave 1 respondents were no longer in the sampling frame) were invited; and 2) new invitees were selected via a stratified random sample with proportionate allocation, on the basis of the physician's sex, specialty group, age group, and rural status.

For all three distinct cohorts across both survey waves (Wave 1 survey, Wave 2 Follow-up, and Wave 2 Supplement), each invited respondent had an equal probability of selection within their respective cohorts. For the Wave 1 Survey and Wave 2 Follow-up, each eligible respondent received an invitation, and thus the probability of selection was a constant 1 for these two cohorts. For the Wave 2 Supplement, 16,900 invitees were selected via a stratified random sample with proportionate allocation (with 180 strata defined by cross-classes of urbanicity, age group, specialty group, and gender). The population proportions within each stratum were computed from the sample frame of eligible physicians as received from Toluna, and the selected sample (n=83,000) was allocated to these strata proportional to the population size of each stratum. This sample design results in a self-weighted sample, where all selected units have an equal probability of selection, and thus no adjustment for selection probability is required.

Response Rates

i) Wave 1: The traditional response rate does not apply, as data collection stopped once we had 6,000 respondents.

ii) Wave 2 Follow-up (repeating respondents): 2,429 Respondents/4,418 Invitees = 55.0% response rate.

iii) Wave 2 Supplement (new invitees): 3,488 Respondents/16,900 Invitees = 20.6% response rate.

Nonresponse adjustment

Differential nonresponse was modeled using data from the Wave 2 (2022) data collection, modeling the probability of a survey response as a function of age, specialty group, gender, and sample cohort (Wave 2 Follow-up vs. Wave 2 Supplemental sample). Since these same data were not available for the 2019 data collection, nonresponse was modeled using the Wave 2 data collection only, with those model- based adjustments also being applied out-of-sample to the 2019 data. The nonresponse adjustment is simply the inverse of the estimated probability for each invitee to respond, so that those invitees that are less likely to respond receive a proportionally higher weight (thus representing themselves and a higher share of those like them), and those that are more likely to respond receive a proportionally lower weight (representing themselves and a lower relative share of those like them).

Calibration

Adjusted weights following the nonresponse adjustment described above were then calibrated to known population distributions in each respective wave (i.e., 2019 survey respondent weights were calibrated to 2019 population distributions, and 2022 survey respondent weights were calibrated to 2022 population distributions, both based on American Medical Association data provided by AAMC) based on cross- classes of age group, specialty group, gender, and International Medical Graduate (IMG) status. This process ensures that weighted distributions of these key domain variables will exactly match the known population distributions for each cross-classed subgroup (these cross-classed subgroups are also called poststrata).

Summary of Weights Development

The University of Michigan Population Dynamics and Health Program team created a survey weight variable for both waves of the NSSP: the Wave 1 2019 survey of physicians and the Wave 2 2022 survey (comprised of the follow-up survey of doctors who responded to Wave 1, aka the "Follow-up", and the supplemental sample of new respondent physicians who did not participate in Wave 1). These weights are designed to allow for stand-alone analysis of each wave separately, or a combined analysis to assess trends from Wave 1 to Wave 2. Both weight variables are a) adjusted for differential non-response modeled using the NSSP Wave 2 (2022) survey invitees and b) calibrated back to known AMA population characteristics specific to each wave (i.e., calibration was done separately for both respective waves).

© 2024 Meeks LM et al. *JAMA Network Open*

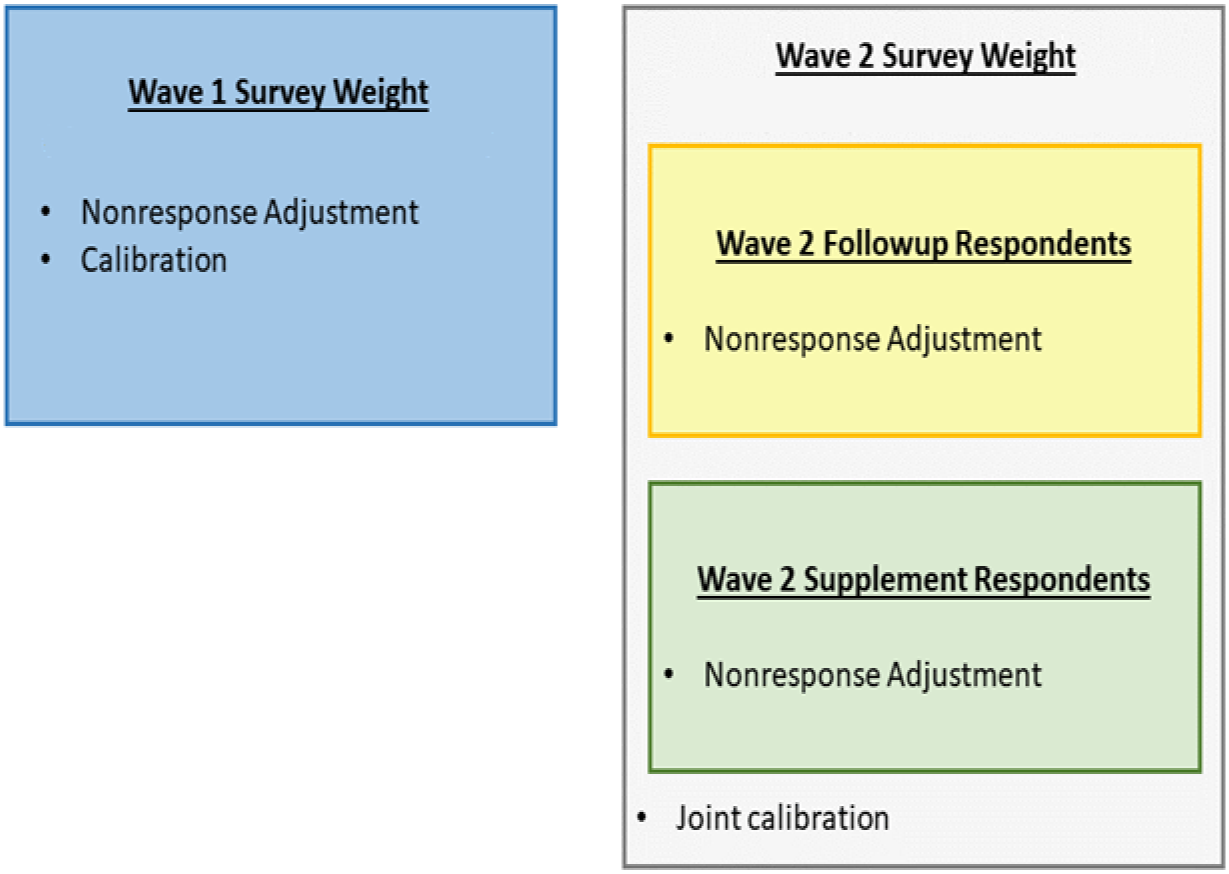

© 2024 Meeks LM et al. *JAMA Network Open*

Demographic and Work Characteristics of Participating Physicians (Count)			
	Physicians without Disabilities	Physicians with Disabilities	Missing Disability Status
	(n=5,575)	(n=185)	(n=157)
**Race/Ethnicity[a]**			
American Indian or Alaskan Native	NA	NA	NA
Asian	1,353	26	48
Black or African American	139	NA	NA
Hispanic, Latino, or of Spanish origin	174	NA	NA
Multiple races/Other	237	17	16
Missing	34	NA	NA
Native Hawaiian or Other Pacific Islander	11	NA	NA
White	3,617	128	81
**Gender identity**			
Woman and trans-woman	1,669	77	46
Man and trans-man	3,873	107	105
Genderqueer or other	33	NA	NA
**Sexual orientation**			
Non-Heterosexual	284	22	18
Heterosexual	5,291	163	139
**Age group (in years)**			
35 or under	437	NA	17
36-55	2,946	72	93
56-75	2,103	99	44
76 or older	89	NA	NA
**Married**			
No	908	46	33
Yes	4,657	139	123
Missing	NA	NA	NA
**Presence of children aged 5 or younger**			
No	4713	173	134
Yes	862	12	23
**Working in hospitals**	1,405	34	36
**Specialty group**			
Medical Specialties	1,165	28	40

© 2024 Meeks LM et al. *JAMA Network Open*

	Physicians without Disabilities	Physicians with Disabilities	Missing Disability Status
Other	1,530	62	46
Primary Care	1,781	74	48
Surgery	1,099	21	23
**International Medical Graduates (IMG)**	857	22	28
**Faculty affiliation**	1,938	74	28
**Working in private setting**	2,172	76	52
Source: AAMC 2022 National Sample Survey of Physicians. Analyses conducted by Z. Nouri.			
Notes: Data representing fewer than 10 participants were not included (NA). Disability is self-reported. Participants were provided with the Americans with Disabilities Act's definition of disability. Respondents who said "I don't know" were excluded from analyses. [a]Race and Ethnicity information were gathered previously by the AAMC and linked to NSSP data. See categories listed below.			

How do you self-identify? You may optionally enter this information on this page. **Please check all that apply.**

☐ Hispanic, Latino, or of Spanish Origin

- ☐ Argentinean
- ☐ Colombian
- ☐ Cuban
- ☐ Dominican
- ☐ Mexican/Chicano
- ☐ Peruvian
- ☐ Puerto Rican
- ☐ Other Hispanic, Latino, or of Spanish Origin ______

☐ American Indian or Alaskan Native

- ☐ Tribal Affiliation:

  ______

☐ Asian

© 2024 Meeks LM et al. *JAMA Network Open*

- ☐ Bangladeshi
- ☐ Cambodian
- ☐ Chinese
- ☐ Filipino
- ☐ Indian
- ☐ Indonesian
- ☐ Japanese
- ☐ Korean
- ☐ Laotian
- ☐ Pakistani
- ☐ Taiwanese
- ☐ Vietnamese

© 2024 Meeks LM et al. *JAMA Network Open*

☐ Other Asian: ______

☐ Black or African American

☐ African American

☐ African

☐ Afro-Caribbean

☐ Other Black or African American ______

☐ Native Hawaiian or Other Pacific Islander

☐ Guamanian

☐ Native Hawaiian

☐ Samoan

☐ Native Hawaiian or Other Pacific Islander Other: ______

© 2024 Meeks LM et al. *JAMA Network Open*
