# Supplementary material for: Burnout Among Physicians With Disabilities
Source: JAMA Netw Open. 2024 May 9;7(5):e2410701. doi: 10.1001/jamanetworkopen.2024.10701 (PMC11082676; doi:10.1001/jamanetworkopen.2024.10701)
Supplement: Supplement 2. — Data Sharing Statement [file jamanetwopen-e2410701-s002.pdf]

Data Sharing Statement

Meeks. Burnout Among Physicians With Disabilities. *JAMA Netw Open*. Published May 09, 2024. doi:10.1001/jamanetworkopen.2024.10701

Data
Data available: Yes
Data types: Deidentified participant data, Data dictionary, Other (please specify)
Additional Information: Only aggregate data and the data dictionary are available in compliance with the IRB unless a data licensing agreement is completed.
How to access data: Michael Dill mdill@aamc.org
When available: With publication

Supporting Documents
Document types: Other (please specify)
Additional Information: Individual's interested in the data much complete a request for data through AAMC data Stewart. mdill@aamc.org
How to access documents: mdill@aamc.org
When available: With publication

Additional Information
Who can access the data: researchers whose proposed use of the data has been approved
Types of analyses: For research purposes
Mechanisms of data availability: with signed data access agreement
Any additional restrictions: N/A
